# Clinical and sonographic improvement of developmental dysplasia of the hip: analysis of 948 patients

**DOI:** 10.1186/s13018-022-03432-7

**Published:** 2022-12-12

**Authors:** Karim Bakti, Vilma Lankinen, Mika Helminen, Jarmo Välipakka, Hannele Laivuori, Anna Hyvärinen

**Affiliations:** 1 Faculty of Medicine and Health Technology, Tampere University, Tampere, Finland; 2 Department of Pediatric Surgery, Turku University Hospital, Savitehtaankatu 5, 20520 Turku, Finland; 3 Faculty of Social Sciences, Health Sciences, Tampere University, Tampere, Finland; 4 Tays Research Services, Tampere University Hospital, Tampere, Finland; 5 Pihlajalinna, Tampere, Finland; 6 Center for Child, Adolescent, and Maternal Health Research, Faculty of Medicine and Health Technology, Tampere University, Tampere, Finland; 7 Department of Obstetrics and Gynecology, Tampere University Hospital, Tampere, Finland; 8 Institute for Molecular Medicine Finland (FIMM), Helsinki Institute of Life Science, University of Helsinki, Helsinki, Finland; 9 Medical and Clinical Genetics, University of Helsinki, Helsinki University Hospital, Helsinki, Finland; 10 Department of Surgery, Mehiläinen Länsi-Pohja Oy, Kemi, Finland; 11 Department of Otorhinolaryngology and Head and Neck Surgery, Oulu University Hospital, Oulu, Finland; 12 Clinical Medicine Research Unit, Medical Research Center, University of Oulu, Oulu, Finland

**Keywords:** DDH, Developmental dysplasia of the hip, Ultrasound, Ultrasonography, Abduction splintage, Pavlik harness

## Abstract

**Background:**

Developmental dysplasia of the hip is a common condition, which varies in severity. Abduction treatment is widely used to correct the development of the hips, but mild forms of DDH can also recover spontaneously. The purpose of this study was to evaluate factors affecting the rate of improvement of developmental dysplasia of the hip, and evaluate any risk factors slowing the process.

**Material and methods:**

The study population consisted of patients diagnosed with DDH in Tampere University hospital in the years 1998–2018. Data were retrospectively collected, and associations between clinical variables and rate of improvement were analyzed. Alpha angles were assessed monthly, and associations between risk factors and improvement of alpha angles were studied. A total of 948 patients were included in the analysis.

**Results:**

More severe first status of the hips was associated with faster improvement in dynamic ultrasound compared to milder DDH in univariate design in first 3 months of age; in the multivariable design, Ortolani positivity was conversely associated with lower alpha angles in 1-month follow-up. Immediate abduction treatment was associated with faster recovery rate compared to delayed abduction or watchful waiting. Female sex and positive family history were associated with slower rate of improvement and lower alpha angles. In multivariable design, female sex, positive family history and treatment strategy remained statistically significant as initiation time of the treatment explained the first found association of clinical hip status and the recovery rate after 2 months of age.

**Conclusion:**

Female sex and positive family history might be independent risk factors for slower recovery in DDH before 6 months of age. These children might need special attention in their follow-up plans and abduction treatment.

## Introduction

Developmental dysplasia of the hip (DDH) includes a spectrum of abnormalities ranging from mild conditions such as laxity of the hip joint to more severe forms of the disease such as full luxation of the hip [1–3]. Incidence of the condition is hard to evaluate since diagnostic criteria and screening methods vary around the world. For this reason, variation in incidence is present in the previous studies [4–7].

Normal development of the fetal hip is a complex process in which appropriate contact between acetabulum and femoral head is crucial. Development continues after birth as femur and acetabulum continue growing [8, 9]. Known risk factors include female sex, pre-natal breech presentation, positive family history, left side, primiparity and clicking hips [10, 11]. Risk factors are thought to be associated in 50–60% of the cases [10].

Diagnosis of DDH is primarily based on clinical findings in infant’s hips after birth. In physical examination, hip joint stability, limb length discrepancies and abduction deficits are assessed. Clinicians use Ortolani and Barlow tests to asses hip joint stability. Ortolani positive sign is detected, when during abduction and flexion of the hip joint femoral head returns to acetabulum making ‘clunk’ sound. Barlow positive hips are more stable, but can be dislocated in clinical evaluation with minor provocation. In the mildest clinical form of DDH hips are a little loose, but do not fully dislocate or subluxate even with provocation. After around 3 months of age, Ortolani and Barlow tests lose their sensitivity and asymmetric hip abduction becomes the most relevant finding [2].

Ultrasound is used in the help of diagnostics and observation of the condition. As the ossification center of the femoral head begins to grow, ultrasound loses its value; thus, at the age of 4–6 months, radiographs are getting more useful to assess DDH [3, 12]. Ultrasound assessment of DDH is based on Graf’s ultrasound classification [13, 14]. Ultrasound features of the disease include lowered alpha angle, beta angle and/or insufficient bony coverage of the femoral head. Radiological maturity of the hip in Graf’s classification is age-dependent. Ultrasound performed during Barlow maneuver tests dynamic stability of the hip. In infants with clinical instability, ultrasound may be performed in 4–6 weeks of age, and it may be used for further evaluation of the condition and as guidance for treatment plans [2, 3, 12]. Universal ultrasound screening is not recommended, as it leads to overtreatment and is not useful in reducing incidence of late cases [12, 15, 16]. Selective secondary radiographic screening in 6 weeks of age is used in children with known risk factors of DDH [12, 17].

Dysplastic changes in infant’s hips may resolve spontaneously; however, conservative treatment has been efficient. Clinical practice guidelines recommend abduction splintage treatment for hips with Ortolani positive DDH [12]. Pavlik harness, Von Rosen splint, Craig splint and Frejka pillow are alternative methods of splintage [18]. Pavlik harness and Von Rosen splint are most used in splinting, and limited evidence supports use of von Rosen splint over other the methods [12, 19, 20]. However, treatment with Pavlik harness is the most widely used method in the world. With Barlow positive hips, it has been proven that waiting the onset of abduction treatment is safe and gives room to spontaneous recovery of mild DDH [21]. Abduction treatment in stable yet dysplastic hips has not been found effective, and in this situation, abnormalities detected in ultrasound can be observed [2, 12, 22–24]. If hips do not improve with conservative treatment, operative treatment may be needed. Early detection and treatment are key factors in successful non-operative management of the disease.

## Objectives

The purpose of this study was to investigate the rate of improvement in DDH and evaluate risk factors affecting the rate. Evaluation of these risk factors helps to understand how improvement in children with DDH could be gained earlier and if there are groups of infants that need special attention in their treatment and/or follow-up plans.

## Materials and methods

Study population consisted of patients diagnosed with DDH according to World Health Organizations International Classification of Diseases and Health Related Problems 9th and 10th revisions (ICD-9 and ICD-10), codes 7543.0–7543.5 (ICD-9) and Q65.0–Q65.5 (ICD-10), treated in Tampere University hospital in the years 1998–2018. Data for the study were retrospectively collected from the patient records. Data regarding family history, newborn birth data, information about the mother regarding the pregnancy, breech presentation, mode of delivery, clinical and radiological status of the infant’s hips and information about the treatment and results of treatment were recorded and analyzed. Patients that only received operative treatment on Tampere University hospital but were initially and postoperatively treated in some other centers were excluded.

Rate of improvement was assessed using alfa angles from sonographic follow-ups. Since the ultrasound is usually performed monthly, we divided the time of improvement in categories: by the end of 1 month, 2 months, 3 months, 4 months, 5 months and 6 months or more. Alpha angles 60 or over were considered normal.

Bivariate analysis of risk factors (clinical hip status, positive family history (defined as one or more first degree relative with DDH), gender, gestation age, primiparity, intra-uterine breech presentation and birth weight) and the rate of improvement were carried out in cross-tabulations separately with every month and chi-square test was used to analyze statistical significance.

First clinical hip status was set by pediatric surgeon or pediatric surgery resident. Hips were ether Ortolani positive, Barlow positive, or Ortolani and Barlow negative, with possible minor laxity of the joint but no dislocation in provocation in otherwise normal hip.

Positive family history was defined as at least one first degree family member (parents or siblings) with the diagnosis of DDH.

In Tampere University Hospital, selective screening is used to patients with DDH suspicion. All the patient with the suspicion of DDH undergo clinical evaluation by pediatric surgeon or pediatric surgery resident within the first week of life, and ultrasound plus clinical control in approximately 3–6 weeks of age. With completely dislocated hips (Ortolani positivity), abduction treatment is started at the first clinical evaluation. With mild DDH (subluxated, minor looseness in the clinical examination), it is up to pediatric surgeon if the abduction treatment is started at the first clinical examination or if the child is taken to watchful waiting protocol. Second examination is at approximately 4 weeks of age together with the dynamic hip ultrasound. Pediatric hip ultrasound examination in our center includes assessment of alpha-angle (based on Graf method) and bony acetabulum coverage of femoral head (according to Terjesen method) as well as dynamic ultrasound evaluation of hip stability during manual axial compression of the femur whilst hip joint is in 90° flexion and slight adduction and the child is lying on his/her side.

In Tampere University Hospital, watchful waiting is one treatment strategy in mild (Ortolani negative) DDH. The decision between the watchful waiting or abduction treatment is made by the pediatric surgeon (not the parents) case by case and is likely to be affected the anamnestic information on the known risk factors (such as DDH in first-degree relatives), in addition to the clinical status and ultrasound imaging. Watchful waiting strategy has been described before in mild DDH [21, 25, 26]. In ultrasound screening of these hips approximately at 4 weeks of age, if the hips are stable in dynamic ultrasound and alpha angles are normal or slightly immature, watchful waiting is continued. With unstable hips, abduction treatment is started. For that reason, we evaluated also associations between the treatment strategy (immediate abduction, delayed abduction after one month of observation and watchful waiting) and rate of improvement in bivariate analysis with chi-square tests.

The concept of confounding factor was assessed in multivariable analysis carried out as a binary logistic regression. The multivariable analysis was used solely as an additional analysis for previous analyses and separately in every month follow-ups. The statistical significance for all tests was set at *p* < 0.05. All analyses were carried out using SPSS version 27.

## Results

A total of 948 patients met the inclusion criteria and were included in the analysis. Of these 686 were girls and 262 boys. Breech presentation at the time of birth was evident in 312 patients. Ortolani positive DDH was diagnosed in 389 patients. Of these, 292 were unilateral and 97 bilateral. All together 226 patients had Barlow positive hip, and 303 patients had both Ortolani and Barlow negative hips. In 662 patients, abduction treatment was performed with either Pavlik harness (*n* = 598) or Frejka pillow (*n* = 62). Late diagnosed (over 3 months of age) DDH was detected in 28 patients. All together 48 (5.1%) patients ended up receiving cast and/or operative treatment, of which 14 were late detected and 3 were teratological dislocations leaving 31 (3.3%) failing the initial treatment. Detailed patient demographics can be seen in Table 1.

**Table 1:** Patient demographics

Patient characteristics (*n* = 948)	*n* (%)
First clinical status of the hip	
Ortolan positive: unilateral	292 (30.8%)
Ortolan positive: bilateral	97 (10.2%)
Barlow positive (Ortolani negative): unilateral	180 (19.0%)
Barlow positive (Ortolani negative): bilateral	46 (4.9%)
Unstable, not dislocatable	303 (32.0%)
Information missing	30 (3.2%)
Treatment	
Immediate abduction treatment	505 (53.3%)
Delayed abduction treatment	157 (16.6%)
Operation and/or casting	48 (5.1%)
Observation (clinical/radiological follow-ups)	266 (28.1%)
Information missing	1 (0.1%)
Gender	
Male	262 (27.6%)
Female	686 (72.4%)
Presentation	
Breech presentation	312 (32.9%)
Cephalic presentation	628 (66.2%)
Information missing	8 (0.8%)
Family history	
Positive	194 (20.5%)
First-degree relative	138 (14.6%)
Negative	382 (40.3%)
Information missing	372 (39.2%)
Parity	
Nulliparous	202 (21.3%)
Primiparous	85 (9.0%)
Multiparous	36 (3.8%)
Information missing	625 (65.9%)
Gestation age	
Term	848 (89.5%)
Premature	38 (4.0%)
Postmature	44 (4.6%)
Information missing	18 (1.9%)
Birth weight	
Normal birth weight	899 (94.8%)
Low birth weight (< 2500 g)	21 (2.2%)
Birth weight (> 4500 g)	19 (2.0%)
Information missing	9 (0.9%)

In 505 of the 662 patients that received abduction treatment, the treatment was initiated early, within the first week of life, after the first clinical examination by pediatric surgeon. In 157 patients, treatment was initiated later after an observation period (Ortolani negative hips). In 19 patients, abduction treatment was followed by operation/casting. Pavlik harness was used in 598 and Frejka pillow in 62 patients.

First clinical status of the hips was associated with higher alpha angles in the 1st-month follow-up, *p* < 0.001. Ortolani positive hips (37.4%) and Barlow positive hips (39.6%) had more often alpha angles 60° or over than hips with only minor looseness (24.6%). After 2 months, there were no more difference between the subgroups (*p* = 0.866), over 73% had normal hips in all the subgroups. After the 6th-month follow-up, initially Ortolani positive hips had more often immature alpha angles (8.2%) compared to Barlow positive (3.6%) and Ortolani and Barlow negative (2.1%) hips, *p* = 0.003.

Female sex was associated with lower alpha angles in the 1st-month (*p* < 0.001), the 2nd-month (*p* > 0.001) and the 3rd-month (*p* = 0.035) follow-ups. Lower percentage of girls had normal hips in each of the follow-ups. See all the results in Tables 2, 3 and 4. In the 6th-month follow-up, there was no more difference in the rate of normalization between sexes (*p* = 0.070).

**Table 2:** Risk factors and alpha angles in the 1st-month follow-up

Risk factor (*n*)	*p* value	Alpha angle in the 1st-month control (degrees)		*p* value in multivariable design
		Under 60	60 or over	
First clinical status	0.002			0.041
Ortolan+ (342)		214 (62.6%)	128 (37.4%)	
Barlow+ (207)		125 (60.4%)	82 (39.6%)	
Minor looseness (256)		193 (75.4%)	63 (24.6%)	
Positive family history	0.597			0.299
Yes (123)		83 (67.5%)	40 (32.5%)	
No (393)		255 (64.9%)	138 (35.1%)	
Sex	< 0.001	0.007		0.009
Boy (228)		127 (55.7%)	101 (44.3%)	
Girl (591)		413 (69.9%)	178 (30.1%)	
Treatment method	< 0.001	< 0.001		< 0.001
IS (458)		271 (59.2%)	107 (40.8%)	
DS (149)		145 (97.3%)	4 (2.7%)	
WW (212)		124 (58.5%)	88 (41.5%)	
Breech presentation	< 0.001	0.002		0.123
No (577)		381 (70%)	163 (30.0%)	
Yes (272)		158 (58.1%)	114 (41.9%)	
First born	0.610			
No (101)		50 (49.5%)	51 (50.5%)	
Yes (165)		87 (52.7%)	78 (47.3%)	
Birthweight	0.529			
Under 2500 g (12)		9 (75.0%)	3 (25%)	
Normal (781)		512 (65.6%)	269 (34.4%)	
Over 4500 g (19)		13 (68.4%)	6 (31.6%)	
Gestational age	0.490			
Preterm (28)		20 (71.4%)	8 (28.6%)	
37–42 weeks (737)		482 (65.4%)	255 (31.6%)	
Over 42 weeks (41)		30 (73.2%)	11 (26.8%)

**Table 3:** Risk factors and alpha angles in the 2nd-month follow-up

Risk factor (*n*)	*p* value	Alpha angle in the 2nd-month control (degrees)		*p* value in multivariable design
		Under 60	60 or over	
First clinical status	0.280			0.220
Ortolan+ (271)		71 (26.2%)	200 (73.8%)	
Barlow+ (167)		41 (26.9%)	122 (73.1%)	
Minor looseness (227)		56 (24.7%)	171 (75.3%)	
Positive family history	0.028			0.016
Yes (102)		33 (32.4%)	69 (67.6%)	
No (332)		72 (21.7%)	260 (78.3%)	
Sex	< 0.001			0.006
Boy (228)		30 (15.4%)	165 (84.6%)	
Girl (591)		144 (29.8%)	339 (70.2%)	
Treatment method	< 0.001			< 0.001
IS (361)		88 (24.4%)	273 (75.6%)	
DS (168)		64 (43.0%)	85 (57.0%)	
WW (149)		22 (13.1%)	146 (86.9%)	
Breech presentation	0.015			0.070
No (577)		130 (28.4%)	327 (71.6%)	
Yes (272)		43 (19.7%)	175 (80.3%)	
First born	0.580			
Birthweight	0.201			
Gestational age	0.201

**Table 4:** Risk factors and alpha angles in the 3rd-month follow-up

Risk factor (*n*)	*p* value	Alpha angle in the 3rd-month control (degrees)		*p* value in multivariable design
		Under 60	60 or over	
First clinical status	0.421			0.527
Ortolan+ (256)		25 (9.8%)	231 (90.2%)	
Barlow+ (165)		17 (10.3%)	142 (89.7%)	
Minor looseness (218)		15 (6.9%)	203 (93.1%)	
Positive family history	0.002			0.002
Yes (97)		16 (16.5%)	81 (83.5%)	
No (321)		21 (6.5%)	300 (93.5%)	
Sex	0.035			0.136
Boy (188)		10 (5.3%)	178 (94.7%)	
Girl (464)		49 (10.6%)	415 (89.4%)	
Treatment method	0.477			0.887
IS (346)		31 (9%)	315 (91.0%)	
DS (131)		15 (11.5%)	116 (88.5%)	
WW (175)		13 (7.4%)	162 (92.6%)	
Breech presentation	0.030			0.049
No (431)		46 (10.7%)	205 (94.5%)	
Yes (214)		12 (5.5%)	385 (89.3%)	
First born	0.840			
Birthweight	0.608			
Gestational age	0.293			

Positive family history of first degree relative was not associated with the rate of normalization in the 1st-month follow-up (32.5% vs 35.1% (*p* = 0.597)), but after 2 months it seemed that those with positive family history had more rarely normal hips (67.6%) than those without family history (78.3%), *p* = 0.028. The difference was statistically significant also in the 3rd-month follow-up (83.5% vs 93.5% (*p* = 0.002)), but no more in the 6th-month follow-up (93.9% vs 96.0% (*p* = 0.344)).

Intra-uterine breech presentation was associated with faster normalization of the hips compared to those in cephalic presentation. In the 1st-month follow-up 41.9% of the children with breech presentation had normal hips compared to 30.0% of those with cephalic presentation, *p* < 0.001. In the 2nd-month follow-up, percentages were 80.3% and 71.6% (*p* < 0.001) in favor of the breech born infants and in the 3rd-month follow-up, percentages were 94.5% and 89.3% (0.030). Difference was also statistically significant in the 6-th month follow-up (97.3% vas 94.1% (*p* = 0.047)).

Treatment strategy was associated with faster recovery rate. Those with initially started abduction treatment and those with only watchful waiting had normal alpha angles more often in the 1st-month follow-up compared to those with delayed start of abduction, *p* < 0.001. The difference between subgroups was most clearly seen in the 2nd-month follow-up. Those in watchful waiting group had highest (86.9%) normalization rate compared to those in initially started abduction (75.6%) and those in delayed abduction (57.0%), *p* < 0.001. In the 3rd-month follow-up, there were no more difference between treatment groups (0.477).

There was no association between correction rate of alpha angles and other studied risk factors (birthweight, first born birth and pregnancy duration), see the *p*-values in Tables 3, 4 and 5.

**Table 5:** Risk factors and alpha angles in the 6th-month follow-up

Risk factor (*n*)	*p* value	Alpha angle in the 6th-month control (degrees)		*p* value in multivariable design
		Under 60	60 or over	
First clinical status	0.003			0.171
Ortolan+ (319)		26 (8.2%)	293 (91.8%)	
Barlow+ (195)		7 (3.6%)	188 (96.4%)	
Minor looseness (242)		5 (2.1%)	237 (97.9%)	
Positive family history	0.344			0.302
Yes (115)		7 (6.1%)	108 (93.9%)	
No (375)		15 (4.0%)	360 (96%)	
Sex	0.070			0.093
Boy (222)		6 (2.7%)	216 (97.3%)	
Girl (550)		32 (5.8%)	518 (94.2%)	
Treatment method	0.186			0.454
IS (428)		27 (6.3%)	401 (93.7%)	
DS (150)		6 (4.0%)	144 (96%)	
WW (194)		5 (2.6%)	189 (97.4%)	
Breech presentation	0.047			0.384
No (506)		30 (5.9%)	476 (94.1%)	
Yes (261)		7 (2.7%)	254 (97.3%)	
First born	0.357			
Birthweight	0.569			
Gestational age	0.597			

Multivariable models were made separately for all follow-up periods. Normalization of alpha angles was used as dependent variable with significant risk factors (first clinical status of the hips, breech presentation, sex, positive family history and treatment strategy) as independent variables. In the multivariable model in the 1st-month follow-up, breech presentation lost its statistical significance, but all the other previously found associations remained statistically significant.

In the multivariable model in the 2nd-month follow-up, first clinical status of the hips (*p* = 0.220) and breech presentation (*p* = 0.070) were no more associated with recovery rate, but sex (*p* = 0.006), positive family history (*p* = 0.016) and treatment strategy (*p* < 0.001) remained statistically significant. In the 3rd-month follow-up, only positive family history (*p* = 0.002) and breech presentation (*p* = 0.049) were statistically significant, and after 6 months, there were no statistically significant associations with nether of the studied variables and recovery rate of alpha angles.

## Discussion

In this study, we found that there are multiple risk factors affecting on the recovery rate in DDH in first 3 months. Some of the known risk factors were associated in slower recovery rate (positive family history and girl sex) and some did not have an effect on the recovery rate (birth weight, pregnancy duration, first born birth) and some were associated with faster recovery rate (breech presentation). In the 6th-month follow-up, all the differences were leveled. We also found that first clinical status of the hip did not have an effect on the recovery rate and in mild DDH watchful waiting strategy seems effective.

It was clear that improvement in our data happened most effectively during the first 3 months. This is likely due to the fact that after birth, acetabular and femoral head remodeling is most rapid during the first weeks of life [1]. It is noted that positive Ortolani test indicates more severe form of DDH when comparing to Barlow positive or mildly loose hips [1–3]. In our data, it seemed that first clinical status of the hip did not have an effect on the recovery rate. Immediate abduction treatment was effective; however, group of children in watchful waiting had the fastest recovery since almost 90% of these children had normal hips in the 2nd-month follow-up. We believe that this is due to excellent remodellation capacity in the first weeks of life combined with successful selection of these children by the pediatric surgeons in our center. According to our data, watchful waiting strategy seems to work in mild DDH; however, it forms a risk of later recovery. Delayed abduction treatment was associated with delayed recovery rate in the 3rd-month follow-up, which, however, does not indicate that delayed abduction treatment would be ineffective in treatment of DDH. Later initiated treatment inevitably leads to delayed improvement. Immediate Pavlik harness treatment in infants with mild DDH (Ortolani negative) is controversial because majority of the infants will improve without treatment, and it seems that it is safe to wait with the initiation of the treatment [27, 28]. Our results give new information as it seems that in mild DDH recovery might delay because of the watchful waiting period. Even if waiting is safe, it is mandatory to inform families of the possibility of more rapid recovery with the immediate abduction treatment. However, further studies are needed to evaluate the effectiveness of the watchful waiting strategy in mild DDH.

Clinical status of the hip and ultrasound appearance are not always congruent, and with ultrasound dysplasia can be detected also in the hips clinically tested as normal [29–31]. However, ultrasound used in screening does not seem to prevent the late cases of DDH and is not associated with improved outcomes when comparing to programs based on clinical appearance of the hip [12, 15, 32, 33]. Radiological classification at birth according to the Graf method has been associated with median age of normalization. Median age of normalization was linear with radiological grade of the hip (Type IIa–Type IV) as more severe forms of DDH gained recovery later [34]. Results of the study are partly in contradiction to our study, which can be partly explained by the fact that treatment initiation indication and treatment method were different from ours.

Female sex is a known risk factor of DDH [10, 11]. In our study, female sex was associated with lower recovery rate compared to male sex (*p* < 0.001) in the 1st, the 2nd- and the 3rd-month follow-up. Sex remained statistically significant in multivariable design. In the previous studies, inconsistent with our finding, male gender has been associated with Pavlik harness treatment failure and slower rate of recovery [35–38]. Our findings, however, indicate that in addition of being a risk factor of DDH, female gender might also be a risk factor of slower recovery of DDH.

Positive family history is a well-known risk factor of DDH [10, 11]. We found that positive family history is associated with slower recovery in first 3 months. This finding remained statistically significant in multivariable design; however, in the 6th-month follow-up, the differentiation was no more statistically significant. This finding gives new information of the effect of positive family history as a risk factor of DDH. Earlier we found that positive family history could also predispose to failure of the Pavlik harness treatment (unpublished manuscript). These findings underline the importance of close follow-up of these patients during the abduction treatment. It seems that genetic factors predispose to severity of DDH and in addition of adding the risk of DDH itself, adding the risk of late recovery and failure of the treatment.

Intrauterine breech presentation was associated with faster recovery rate in the first 3 months of age. However, in multivariable design, it seemed that the found association was explained with other variables. Association was only barely statistically significant in multivariable design in the 3rd-month control. We believe that this is due to two reasons. Earlier we found (unpublished manuscript) that breech presentation might predispose to Ortolani positive dislocation, which means that the initial abduction treatment is started. We also believe that clinicians might start abduction treatment initially more easily in breech born infants, even if the hips are only mildly loose (Ortolani negative) compared to those children without this risk factor. The initial abduction treatment, however, seems to explain the faster recovery rate of breech born children. Our finding indicates that breech born children are to recover well with correct treatment and do not have a risk of delayed recovery. We think that this is due to breech presentation being purely mechanical risk factor of DDH, without any additional genetic or hormonal factors affecting on the condition.

For other risk factors assessed in this study (parity, birth weight and gestation age), no associations with the rate of improvement of alpha angles were found.

Our study has some limitations that should be addressed. The data were collected retrospectively, and the earliest cases dated back over 20 years. Although the sample size was substantial, data were not inclusive for all patients. Data of parity were missing for most of the patients, and data of family history were also inadequate. Due to this factor, our results considering these risk factors might not be adequate. However, we still found that positive family history might predispose to delayed improvement, despite the incomplete data of this part. In our center, ultrasound evaluation includes measurements of alpha angles (according to Graf’s criteria) and bony coverage of acetabulum (according to Terjesen method) as well as dynamic evaluation of hip stability during provocation. Our radiologists have not reported beta angles, and due to that we could not classify hips further according to Graf’s criteria. However, normal hips were considered to have alpha angles over 60° (at any age), which is comparable to Graf’s criteria, and we still found clear associations of risk factors and recovery rate of DDH. Despite the differences to Graf’s ultrasound evaluation, our center had only 48 (5.1%) children needing casting or operation, which of 14 were late diagnosed cases and 3 were teratological dislocations leaving only 31 (3.3%) children failing the initial treatment in 20 years of time. Even though the concept of confounding factor was taken in account in the multivariable analysis, the multivariable analysis itself was not perfectly fit for our data because of the incomplete data of family history, leaving some of the patients out of the multivariable analysis. However, our findings still were in line with the first found associations in the univariate designs. In Tampere University Hospital, where the study data originate, Pavlik harness is used for abduction treatment. Previously Frejka pillow was also used. Our results on the treatment should be considered with precautions, keeping in mind that abduction treatment methods vary according to location.

## Conclusion

Of the known risk factors of DDH, female sex and positive family history were associated with slower rate of improvement in first 3 months of age. Breech born infants seem to recover fast with correct treatment, and breech presentation does not form a risk of slower recovery of DDH. Group of children with Ortolani negative DDH recovered fast in watchful waiting period. Further clinical trials are needed to confirm these findings and analyze further the impact of spontaneous recovery potential of mild DDH to the benefits of early abduction treatment.

## Data Availability

The datasets generated and/or analyzed during the current study are not publicly available due to patient privacy and confidentiality but are available from the corresponding author on reasonable request.

## Abbreviation

DDH: Developmental dysplasia of the hip
